# Impact on Some Soil Physical and Chemical Properties Caused by Metal and Metallic Oxide Engineered Nanoparticles: A Review

**DOI:** 10.3390/nano13030572

**Published:** 2023-01-31

**Authors:** Jonathan Suazo-Hernández, Nicolás Arancibia-Miranda, Rawan Mlih, Lizethly Cáceres-Jensen, Nanthi Bolan, María de la Luz Mora

**Affiliations:** 1 Center of Plant, Soil Interaction and Natural Resources Biotechnology, Scientific and Biotechnological Bioresource Nucleus (BIOREN-UFRO), Universidad de La Frontera, Avenida Francisco Salazar 01145, Temuco 4780000, Chile; 2 Department of Chemical Sciences and Natural Resources, Universidad de La Frontera, Avenida Francisco Salazar 01145, Temuco 4811230, Chile; 3 Faculty of Chemistry and Biology, University of Santiago of Chile (USACH), Santiago 8320000, Chile; 4 Center for the Development of Nanoscience and Nanotechnology, CEDENNA, Santiago 9170124, Chile; 5 Institute of Bio- and Geosciences, Agrosphere (IBG-3), Forschungszentrum Juelich (FZJ), 52425 Juelich, Germany; 6 Physical & Analytical Chemistry Laboratory (PachemLab), Nucleus of Computational Thinking and Education for Sustainable Development (NuCES), Center for Research in Education (CIE-UMCE), Department of Chemistry, Metropolitan University of Educational Sciences, Santiago 776019, Chile; 7 School of Agriculture and Environment, The University of Western Australia, Perth, WA 6009, Australia; 8 The UWA Institute of Agriculture, The University of Western Australia, Perth, WA 6009, Australia

**Keywords:** nanoparticles, soil properties, environment, emerging pollutants

## Abstract

In recent years, the release of metal and metallic oxide engineered nanoparticles (ENPs) into the environment has generated an increase in their accumulation in agricultural soils, which is a serious risk to the ecosystem and soil health. Here, we show the impact of ENPs on the physical and chemical properties of soils. A literature search was performed in the Scopus database using the keywords ENPs, plus soil physical properties or soil chemical properties, and elements availability. In general, we found that the presence of metal and metallic oxide ENPs in soils can increase hydraulic conductivity and soil porosity and reduce the distance between soil particles, as well as causing a variation in pH, cation exchange capacity (CEC), electrical conductivity (EC), redox potential (Eh), and soil organic matter (SOM) content. Furthermore, ENPs or the metal cations released from them in soils can interact with nutrients like phosphorus (P) forming complexes or precipitates, decreasing their bioavailability in the soil solution. The results depend on the soil properties and the doses, exposure duration, concentrations, and type of ENPs. Therefore, we suggest that particular attention should be paid to every kind of metal and metallic oxide ENPs deposited into the soil.

## 1. Introduction

Engineered nanoparticles (ENPs) are materials intentionally produced with a particle size between 1 and 100 nm in at least one dimension, which are present in the form of a nanowire, spherical, nanotubes, and nanorods [1]. ENPs are divided into five classes; based on carbon, zero valence metal, metallic oxide, quantum points, and dendrimers [2]. These nanoparticles possess i) novel physicochemical characteristics such as a high surface area for reactions and interactions, and ii) exceptional optical, magnetic, and electrical properties compared to their bulk counterparts [3,4,5]. As a consequence of those advantages, in the last decade, the production and subsequent incorporation of ENPs in products such as cosmetics, clothes, pigments, industrial coatings, plastic additives, semiconductors, textiles, and antibacterial agents have increased considerably [6,7]. Currently, there are more than 1800 products containing ENPs in the market [8], and worldwide production of ENPs is expected to reach $125 billion by 2024 [9]. Therefore, nanotechnology is a science that has had and will continue to have great importance in improving the quality of life for humans [10]. However, this also means that the type and volume of ENPs released into the environment will increase [11].

Among the different ENPs that exist in the market, metal (e.g., Au, Al, Ag, Fe, and Cu) and metallic oxide (e.g., TiO_2_, ZnO, Al_2_O_3_, Fe_3_O_4_, Fe_2_O_3_, NiO, CuO, Cu_2_O, and CeO_2_) ENPs are those with the greatest probability of being deposited in soils and in particular for agricultural use [12,13,14]. This is because they have antimicrobial properties, or the elements released from ENPs are nutrients for plants. Consequently, they can be incorporated and/or used as pesticides, insecticides, herbicides, fungicides, and fertilizers [15,16,17,18]. Various studies have shown that applying metal and metallic oxide ENPs are a promising alternative to treat infections in plants and increase plant development without impairing productivity by reducing the number of agrochemicals added to the soil. In this sense, it is estimated that the concentration of metal and metallic oxide ENPs deposited in agricultural soils could increase from 30 pg kg^−1^ in 2017 to 10 g kg^−1^ in 2050 [19]. Therefore, monitoring their presence in this non-renewable natural system is essential.

Once the metal and metallic oxide ENPs are in contact with the soil system, they can leach into groundwater or suffer biological, chemical, and photochemical transformations (e.g., homo/heteroaggregation, oxidation, dissolution, and precipitation) [20,21,22]. As a result, ENPs can cause changes in the biological (e.g., mesofauna, macrofauna, and microbiota), physical (e.g., hydraulic conductivity, porosity, texture, bulk density, aggregation), and chemical (e.g., cation exchange capacity (CEC), electricity conductivity (EC), redox potential (Eh), pH, dissolved organic matter (DOM), and organic matter (OM) content) properties of the soil [16,20,23,24,25]. In fact, due to their small particle size, ENPs can interact with plant nutrients, like phosphorus (P), affecting their availability in the soil solution [26,27,28].

Various reviews have been published about the transport, fate, and transformations of ENPs in soils and their effect on the abundance and diversity of microorganisms and on plant growth to date [21,29,30,31]. By contrast, there are only a few reviews about the effect of different metal and metallic oxide ENPs on the soil abiotic properties. One of the most recent reviews was published by Sun et al. [13], who concluded that an increase in the concentration of ENPs in soils might affect soil biochemical properties.

Therefore, there is a need to link the information about the effect of metal and metallic oxide ENPs on soil systems through a review. This review aimed to present the effects that different types of metal and metallic oxide ENPs cause on the physical and chemical properties of the soil. This review will help to understand the impact of ENPs on health and the balance in the soil system.

## 2. Soil Health and Quality

**Figure 1:**
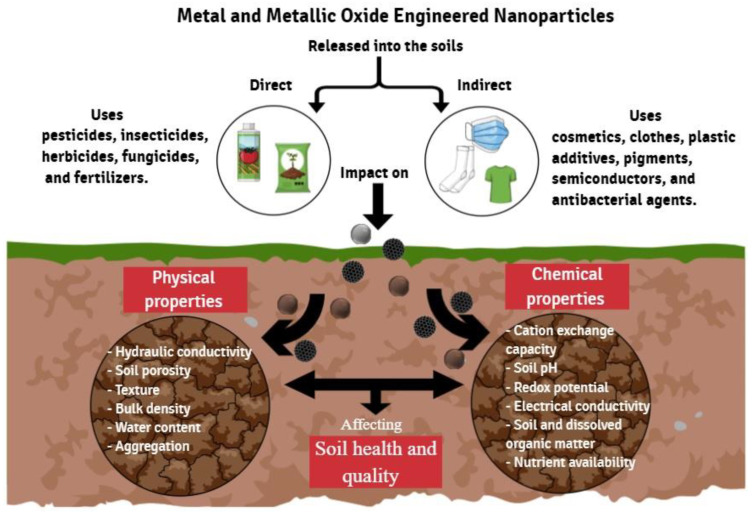
Summary of the impact of metal and metallic oxide engineered nanoparticles on physical and chemical soil properties.

The physical and chemical properties of soil significantly influence soil health and quality. Therefore, the possibility that those factors decrease in the face of the effect of the millions of tons of ENPs accumulated annually is very high [19]. It is known that ENPs can suffer different processes and transformations in the soil system. In contrast, the impact on abiotic properties of soil and biogeochemical cycles has gone practically unnoticed due to the limited and scattered evidence [20]. However, more recently, studies have increased, revealing that physical and chemical properties are affected by the deposition of ENPs [13]. A list of studies investigating the impacts of ENPs on various abiotic soil properties is provided in Table 1 and a summary is provided in Figure 1.

## 3. Effect of ENPs on Soil Properties

### 3.1. Physical Properties

The physical and mechanical soil properties, including structure, bulk density, porosity, permeability, texture, temperature, moisture, and others, are relevant because they are correlated with the productivity of different plants and root growth [33]. Studies on physical properties have shown that ENPs such as Fe_3_O_4_, ZnO, MgO, SiO_2_, and TiO_2_ [33,34,54] can increase hydraulic conductivity and soil porosity and reduce the distance between soil particles (Table 1). As a consequence of this, ENPs aid in forming a more rigid matrix, favoring the increase in agricultural productivity and producing a safer environment and a healthier life. Concerning this, Bayat et al. [33] investigated the effects of the application of Fe_3_O_4_ and MgO ENPs (three doses of 1, 3, and 5% *w/w*) on total porosity, mean weight diameter aggregate, volumetric water content, penetration resistance, and saturated hydraulic conductivity during incubation periods of 40 and 100 days. They concluded that only MgO ENPs improved the soil’s physical and mechanical properties due to their excellent adhesiveness, specific surface, activity, and reaction capacity.

Similarly, Bayat et al. [55] added MgO and Fe_3_O_4_ ENPs (<100 nm) into calcareous loamy soil after being subjected to various stresses. They found that MgO ENPs caused a decrease in soil bulk density compared to the effect produced by Fe_3_O_4_ ENPs. The reduction in density provides better aeration and penetration of roots in the soil. The difference was related to the smaller particle size of MgO ENPs (however, the specific size for both ENPs was not shown) compared to Fe_3_O_4_ ENPs. It was also found that MgO NPs improved soil structure, increased porosity, and reduced bulk density, whereas Fe_3_O_4_ ENPs only increased the tensile strength of the aggregates by strengthening the bonds between Fe and soil particles [55]. In addition, the physical properties of soil can be affected by the concentrations and particle sizes of ENPs. Komendová et al. [24] observed an increase in the strength of the water molecule bridges and the structural rigidity of the soil after using Pt ENPs of 3 nm in concentrations of 0.1, 1, and 10 μg by 300 mg^−1^ soil. However, at concentrations between 100 and 1000 μg by 300 mg^−1^ soil, they decreased the water content retained SOM. In the same way, Fe ENPs, with a smaller particle size than the pores of clay soil, managed to leach through it, but over time the soil pores could become clogged and consequently reduce the hydraulic conductivity due to the formation of aggregates [35].

### 3.2. Chemical Properties

#### 3.2.1. Cation Exchange Capacity

The CEC of soil depends on the surface charge and surface area. In agricultural soils, CEC is a relevant parameter because it is an indicator of the ability of the soil to adsorb nutrients, in other words, of its fertilization [10,23]. A few studies have shown information on CEC in the presence of ENPs. De Souza et al. [42] showed that adding 2000 mg L^−1^ of Fe_3_O_4_ ENPs into a clay-textured soil caused an increase a 17% in the CEC (49.2 meq 100 g^−1^) compared to the control soil (42.2 meq 100 g^−1^). Meanwhile, Baragaño et al. [56] treated technosol soil with Fe ENPs in a 97.5:2.5 soil–ENPs ratio and obtained null variations in the CEC values.

Several researchers have stabilized metal and metallic oxide ENPs with organic molecules or immobilized and blended them with substrates. In this regard, the type of material used is highly relevant to changes shown by CEC values. Das et al. [10] synthesized Ag ENPs through green synthesis using an extract plant leaf (*Thuja occidentalis*) (GSENPs) and conventionally synthesized silver engineered nanoparticles (CSENPs). Both MPs were added in doses of 20, 25, 50, and 100 mg kg^−1^ to an alluvial soil, causing an increase in CEC between 1.01 and 3.35 times for CSENPs and between 1.27 and 3.47 times for GSENPs compared to control soil. This was because both ENPs caused an increase in soil porosity between 1.12 and 1.26 times for CSENPs and between 1.07 and 1.31 times for GSENPs, generating an improvement in the rate of stabilization of OM in soil. In addition, Ag GSENPs generated a change in the soil ionization, increasing the reactive surface and the net negative charge. Likewise, an increase in CEC has been reported between 9.4% and 64.1% for plowed soil with a dose range between 0.05–1.60%, w/w of Fe_3_O_4_ ENPs-biochar compared to soil without ENPs [57].

Similarly, adding a blend of Fe ENPs-compost-biochar composite to the soil from northern Spain after 15 and 75 days increased the CEC between 7 and 6.8 times, respectively, compared to the control soil [58]. As a control treatment, the authors added a sand-Fe ENPs mixture to the soil, and the CEC values obtained were similar to the control soil. Thus, they concluded that changes in the CEC were not associated with ENPs but were caused by biochar.

#### 3.2.2. Soil pH

Soil pH is a factor that is directly related to soil fertility and health [59]. The pH values obtained in soils with ENPs are diverse. Studies carried out on different soils with ENPs of Ag [10], phytogenic iron oxide [60], ZnO [61], CuO [52], Fe_3_O_4_ [62], and ZnO and CuO [28] have shown a slight increase in pH values (Table 1). Gao et al. [52] used 10 mg kg^−1^ of CuO ENPs in sandy soil and determined that the pH ranged from 4.9 to 5, which is similar to the pH value of the control soil. Meanwhile, with 40 mg kg^−1^ CuO ENPs the pH increased from 5.1 to 5.4. This increase was less than expected, suggesting that the soil buffer capacity limited the increase in soil pH. The mechanism involved in the slight increase pH was the hydrolysis of CuO ENPs (it can also be used for ZnO ENPs) caused by the water contained in the soil pores and represented by Equations (1) and (2).

(1) $${\;\mathrm{CuO}}_{\left(S\right)}+H_{2}O_{\left(I\right)}↔\mathrm{Cu}{\left(\mathrm{OH}\right)}_{2\left(S\right)}$$

(2) $$\mathrm{Cu}{\left(\mathrm{OH}\right)}_{2\left(S\right)}+2H_{\left(\mathrm{aq}\right)}^{+}↔{\;\mathrm{Cu}}^{2+}+2H_{2}O_{\left(I\right)}$$

Fe ENPs have been widely used in studies of nanoremediation [63,64]. Therefore, a high amount of those ENPs can be deposited into natural soil systems. In this context, adding 10 mg g^−1^ Fe ENPs to soils from Hangzhou increased the pH between 0.10–0.40 units [37]. These results were attributed to the oxidation process of Fe ENPs in the environment, represented by Equations (3) and (4) [65,66].

(3) $${\mathrm{Fe}}_{\left(s\right)}^{0}+4H_{\left(\mathrm{ac}\right)}^{+}+{\;O}_{2\left(\mathrm{aq}\right)}\to \;2{\mathrm{Fe}}_{\left(\mathrm{aq}\right)}^{2+}+2H_{2}O$$

(4) $${\;\mathrm{Fe}}_{\left(s\right)}^{0}+2H_{2}O\;\to \;2{\mathrm{Fe}}_{\left(\mathrm{aq}\right)}^{2+}+H_{2\left(g\right)}+2{\mathrm{OH}}_{\left(\mathrm{aq}\right)}^{-}$$

Subsequently, the Fe^2+^ released can be oxidized according to Equations (5) and (6) [67].

(5) $$2{\mathrm{Fe}}_{\left(\mathrm{aq}\right)}^{2+}+2H_{\left(\mathrm{ac}\right)}^{+}+1/2O_{\left(2\right)\left(\mathrm{aq}\right)}\to 2{\mathrm{Fe}}_{\left(\mathrm{aq}\right)}^{3+}+H_{2}O_{\left(l\right)}$$

(6) $$2{\mathrm{Fe}}_{\left(\mathrm{aq}\right)}^{2+}+2H_{2}O_{\left(l\right)}\to 2{\mathrm{Fe}}_{\left(\mathrm{aq}\right)}^{3+}+{\;H}_{2\left(g\right)}+2{\mathrm{OH}}_{\left(\mathrm{aq}\right)\;\;\;}^{-}$$

On the other hand, there are studies where the changes in soil pH have been due to an indirect action of ENPs. In the study carried out by Zhang et al. [68], 100 mg kg^−1^ of Ag ENPs were added to soils in the absence or presence of cucumber (*Cucumis sativa*) plants. After 60 days, it was determined that the pH increased from 5.28 to 5.33 and from 5.18 to 5.26 for soil with and without the plant, respectively, which was associated with the alteration of metabolites in the soil by exposure to Ag ENPs.

Some studies have reported a slight decrease in soil pH values after the incorporation of ENPs. Duncan and Owens [47] found that after adding 500 mg kg^−1^ of CeO and TiO_2_ ENPs to Australian soils, the pH values decreased between 0.1 and 0.3. On the other hand, Zahra et al. [45] showed that adding 50 and 100 mg kg^−1^ of TiO_2_ ENPs to soil decreased the rhizosphere pH from 7.3 to 7.1. These authors did not provide information on the mechanism involved in the pH decrease because it was not the objective of their study. In the presence of 2, 4, and 6 g kg^−1^ of Fe, Fe_2_O_3_, and Fe_3_O_4_ ENPs in red soil, the pH decreased between 0.4 and 0.8 units on day 7 and in the Wushan soil between 0.60 and 1.10 units on the day 2 compared to the control soils. The acidification of both soils was related to the hydrolysis of Fe^3+^ ions [51].

The variation in soil pH by the presence of ENPs depends on matrix properties and the type of ENPs. After adding Fe ENPs to acidic soil, Mar Gil-Díaz et al. [39] found that the pH increased from ≈5.30 to 7.60, while for calcareous soil, the pH value was nearly 8.0 with and without ENPs. In the calcareous soil, pH values showed no variation, which was explained by the high carbonate content, and their capacity to buffer soil pH variations (CaCO_3_ = 5.6% for calcareous soil and 0.15% for acid soil) [39]. After the incorporation of CuO ENPs at 10, 100, and 1000 mg kg^−1^ into two soils from Huizhou, Shi et al. [46] found that ENPs caused a significant pH increase in soil with less OM content. In addition, Cu^2+^ ions released from ENPs into the solution progressed towards the formation of more stable species such as Cu_2_S and Cu(OH)_2_, which also increased the soil pH.

#### 3.2.3. Redox Potential

The soil Eh represents the oxidation-reduction reactions and depends on the oxygen (O_2_) concentration, precipitation, temperature, and OM content [28]. Eh in agronomy is an essential parameter due to influences in the functioning of the soil–plant–microorganism system and the solubility of nutrients and contaminants. Studies of Eh soils without and with ENPs have received scant attention due to the interdependence between pH and Eh and the difficulty of reproducing, comparing, and interpreting the results obtained [69]. In soils, most metal ENPs tend to oxidize. In other words, they lose electrons, which are captured by substances from the external environment and, as a result, change the Eh values. Fe ENPs, due to their reduction potential (E^0^, −0.41 V), are easily oxidized by O_2_ of the environment, forming Fe^2+^/Fe^3+^ species [70]. Those cations can form a superficial shell-core in ENPs formed by different iron oxides [71]. As a consequence of the redox process, Fe ENPs has been widely used to degrade organic pollutants such as chlorinated methane, benzenes, organochlorine pesticides, chlorinated phenols, and to reduce inorganic pollutants such as As^V^, Se^VI^, Cr^VI^, Pb^2+^, Hg^2+^, and Zn^2+^ [70]. Vítková et al. [72] investigated the effect of Fe ENPs application on Zn and As availability in the rhizosphere of contaminated soils and found that Eh for the control soil ranged between 310–410 mV. After incubating the soil with As (15.9 g kg^−1^), and Fe ENPs at 1 wt%, the Eh increased after a week, but after 5 weeks, it decreased. By contrast, when they added Zn (4.1 g kg^−1^) and Fe ENPs at 1 wt%, there was an increase in Eh from 400–460 mV. The difference was associated with the presence of redox-active elements such as As, Fe, Mn, O_2_, and NO_3_^−^, and their rapid reaction with Fe ENPs. The authors concluded that the variation in Eh values was highly dependent on doses of ENPs and incubation time, which was associated with the amount of reactive mass of ENPs.

In the case of metallic oxide ENPs, although elements are oxidized, they can influence the modification of the soil Eh. For instance, mixed-valence of Fe_3_O_4_ ENPs, uncoated and with dimercaptosuccinic acid (DMSA) coating, were added in natural wetland organic-rich soil. The Eh values obtained with ENPs were between 350 and 440 mV, while for the control soil they fluctuated between 417 and 457 mV [62]. Environmental conditions, such as aerobic and anaerobic systems as well as flooding conditions, are determining factors in the variations of Eh values. Studies on rice growth have related the variations in Eh values with the changes and transformations of ENPs [44,73]. Peng et al. [44] determined that 1000 mg kg^−1^ of CuO ENPs during the maturation stage of the rice caused an Eh decrease of 202.75 mV compared to the control system. The reason was that ENPs have catalytic properties; therefore, they can accelerate the generation of organic reducing substances. On the other hand, Peng et al. [61] reported that the addition of treatments of 50, 100, and 500 mg kg^−1^ ZnO, CuO and CeO_2_ ENPs, increased the Eh values from −222,67 mV (control soil) to −130 mV–−75 mV for all treatments. In particular, Eh values proved to be highly influenced by doses of ENPs. In addition, they evaluated flood conditions for 30 days, where the Eh value decreased due to the presence of ENPs. This behavior was occasioned by the depletion of O_2_ in the soil due to microbial respiration and by producing organic reducing substances through OM decomposition [61]. Conversely, Zhang et al. [28] determined that CuO and ZnO ENPs in flooding conditions in a paddy soil increased Eh values by about 20~30 mV, which was explained because, in flooding conditions, ENPs can consume the reducing substance (H^+^) [28].

Other factors that influence soil Eh values are related to the presence of stabilizing agents such as proteins, humic acid, and chloride [74], and toxic effects of ENPs on soil microorganisms, which have been analyzed in various reviews [9,20,75,76]. In the case of Ag ENPs, which have antimicrobial properties, it has been reported that the variations of Eh values have been a consequence of the decrease in soil microbiology [28].

#### 3.2.4. Electrical Conductivity

Soil electrical conductivity (EC) is a measure of total soluble salts. Various studies using different soils have reported that ENPs such as ZnO, CuO, and CeO_2_ [44], CuO, TiO_2_, ZnO [61], CuO [28], and TiO_2_ [45] have increased the EC values. In particular, Zahra et al. [45] found that with 50 and 100 mg kg^−1^ of TiO_2_ ENPs, the EC values of the rhizosphere increased from ≈0.36 µS cm^−1^ to 0.60 µS cm^−1^ and 0.52 µS cm^−1^, respectively. The explanation was associated with the dissolution process of ENPs, which caused an increase in the number of cations in the solution. In the experiment carried out by García-Gómez et al. [77], the biological effect of ZnO ENPs on earthworms in agricultural soils was evaluated. They found at day 0 that the EC values were 284 µS cm^−1^ and 216 µS cm^−1^ for the soil control and system ZnO ENPs + soil, respectively, while after 35 days, the EC value for the control soil was 314 µS cm^−1^, and for the system ZnO ENPs + soil was 283 µS cm^−1^. The increase in the EC values obtained with the exposure time was related to the solubilization of ENPs. There are also studies where a decrease has been reported in EC values of soil due to the presence of ENPs. For example, in a study conducted by García-Gómez et al. [78] in soils located near Madrid, the EC decreased by ZnO ENPs, which was associated with the capacity of ENPs and/or the cations released from ENPs to combine with cations or anions contained in the soil. Similarly, after applying ZnO and SiO_2_ ENPs (2% and 6%, respectively) in saline soils, Kheir et al. [34] reported a slight decrease in EC values compared to the control soil. However, in this case, the reasons involved in the EC values obtained were not explained.

There are studies where the addition of Ag ENPs has generated null effects on EC values, which has been mainly related to the low doses of ENPs [79,80]. Ag ENPs, in particular, are highly stable, so during laboratory experiments, it is unlikely they undergo oxidation processes and release cations into the soil solution [81]. Fabrega et al. [82] found that in a concentration range between 2–2000 µg L^−1^ of Ag ENPs, less than 2% of ENPs were solubilized. Likewise, the stability of ENPs was increased when stabilizers such as PVP [81] or citrate [83] were used.

#### 3.2.5. Soil and Dissolved Organic Matter

The most productive agricultural soils contain a high percentage of OM. Several studies have evaluated the effect of OM (SOM and DOM) on the toxicity, transformations, and mobility of ENPs [25,84,85,86]. It is known that DOM can be adsorbed on the surface of ENPs, improving their stability and preventing the release of ions from them [87,88], which reduces the toxicity of ENPs [89]. This is due to the mechanism that exists between ENPs being electrostatic and/or steric repulsion, which decreases the aggregation rate and the residence time of ENPs in the soils, thereby increasing the possibility of moving towards other natural systems such as groundwater and rivers [90].

On the other hand, the effects caused by ENPs on DOM are diverse. For example, in a study conducted by Lin et al. [60] in soils taken near a mine in Hunan, which was treated with 9% phytogenic iron oxide nanoparticle (PION), it was found that the DOC increased between 1.54 and 2.81 times compared to the system without ENPs. These results were related to the nature of PION because *Excoecaria cochinchinensis*, which was used as a reducing agent for ENPs, contains a large number of organic biomolecules. These molecules can be easily decomposed/degraded by soil microorganisms [60]. On the other hand, Zahra et al. [45], after investigating the effect of concentrations of 50 mg kg^−1^ and 100 mg kg^−1^ of TiO_2_ ENPs in soils from China, found a dual behavior since 50 mg kg^−1^ of TiO_2_ ENPs reduced the DOC by 11.6%, but with 100 mg kg^−1^ of TiO_2_ ENPs the DOC increased by 25.5%. Specifically, the increase in DOC was explained by two reasons: (i) root–microbe interactions can stimulate roots to secrete a greater amount of exudate, and (ii) roots with a high quantity of ENPs can cause stress to the plant, inducing the release of low molecular weight substances (LMWS) such as oxalate, acetate, and malate [45,91].

In the soil, microorganisms are responsible for regulating OM decomposition and nutrient mineralization. However, ENPs due to catalytic and/or antimicrobial properties or as a consequence of the decrease in soil pH can decrease SOM content [47,92]. Some metal and metallic oxide ENPs that have shown those properties are Ag, Fe, TiO_2_, ZnO, and CuO [46,93]. Rashid et al. [93] investigated the effect of 1000 mg kg^−1^ of ZnO ENPs on carbon and nitrogen mineralization of *Phoenix dactylifera* leaf litter in sandy soil. They found that ENPs reduced carbon (130%) and nitrogen (122%) mineralization efficiency from date palm leaf litter in sandy soil. The reason was due to the soil with ENPs having a lower microbial biomass carbon and the number of colonies of heterotrophic cultivable fungi and bacteria. By contrast, Shi et al. [46], after flooding a paddy soil for 60 days with a concentration of 1000 mg kg^−1^ of CuO ENPs, found that the mineralization of OM was accelerated, as well as increasing the Fe reduction process by increasing the Fe^2+^ content by 293%. These results were associated with the catalyst properties of ENPs.

On the other hand, null changes in total OM content have been determined using concentrations of 10 and 100 mg kg^−1^ of CeO_2_, Fe_3_O_4_ and SnO_2_ NPs [94], 1000 mg kg^−1^ of ZnO ENPs [93], 10 and 100 mg kg^−1^ of Ag ENPs [95], and 1% (w/w) Fe_3_O_4_ and CuO ENPs: soil [23]. The reason was related to the low amount of added ENPs. Specifically, Ben-Moshe et al. [23] added Fe_3_O_4_ and CuO ENPs to a Red Sandy clay loam and Rendzina soil.

#### 3.2.6. Nutrients Availability

All plants require macronutrients like P, nitrogen (N), and potassium (K) for growth. In particular, P in the environment exists as H_3_PO_4_, H_2_PO_4_^−^, HPO_4_^2−^, and PO_4_^3−^, the dissociation constants of which are: pK_1_ = 2.21, pK_2_ = 7.21, and pK_3_ = 12.67, respectively [96]. In agricultural soil, phosphate in H_2_PO_4_^−^ and HPO_4_^2−^ helps plant growth and microorganisms, whose bioavailability may be affected by the deposition of pollutants, including ENPs [97]. Various studies conducted in aqueous systems have reported that phosphate can be adsorbed on ENPs like CeO_2_ [98], magnetic iron oxide [99], Fe [100,101], ZnO [13], Fe/Cu [102], and TiO_2_ [103]. It has been established that there is a chemical interaction between phosphate and active sites of different ENPs; the bonding is irreversible. In addition, those studies suggest that cations released from ENPs can form complexes and/or precipitates with phosphate. Although those investigations were not carried out in soils, they could be an approach to what could happen in the soil matrix. In fact, in the study carried out by Moharami and Jalali [104], they found that Al_2_O_3_ and Fe_3_O_4_ ENPs increased phosphate adsorption in calcareous soil. In addition, the presence of ENPs favored the transfer of phosphate from the HCl-P fraction to the Res-P and NaOH-P. Based on this, they concluded that the bioavailability of phosphate decreases due to the addition of ENPs [104]. In the same way, Koopmans et al. [105], using ferrihydrite of a size between 2–3 nm and a surface area of about 5.4 m^2^ g^−1^, determined that the phosphate concentration in the 0.01 M CaCl_2_ soil extracts decreased. Recently, Suazo-Hernández et al. [26,27] determined that L-ascorbic acid-coated Cu or Ag ENPs increased phosphate adsorption in an Andisol and its fractions. Particularly, in Suazo-Hernández et al. [26], using the Langmuir model, they concluded that by increasing Ag or Cu NPs content from 0 to 5%, the q_max_ values of Pi for the Andisol increased by 46% and 54% following the addition of Cu or Ag ENPs, respectively. These results were attributed to a decrease in soil solution, which is due to the coating of ENPs with L-ascorbic acid and probably some dissolved L-ascorbic acid. This study is relevant because Cu or Ag ENPs are being used as nano-pesticides, so large amount of ENPs can be deposited in soils.

One of the ENPs most likely to reach agricultural soils is ZnO because Zn is a necessary micronutrient for plants, and can therefore be incorporated into soils through agrochemicals [106]. The interaction between PO_4_^3−^ and ZnO ENPs is related to the release of ions from ENPs. Subsequently, they can form a micrometer scale crystalline zinc phosphate and a nanoscale amorphous Zn_3_(PO_4_)_2_ shell [107]. Likewise, Zn is one of the essential structural components of the enzymes phytase and phosphatase which participates in the mobilization of native P. Studies carried out by Verma et al. [48] found that ZnO ENPs increased the secretion of P mobilizing enzymes and consequently increased the concentration of phosphate bioavailable in the soil. Thus, the effect of ENPs on the bioavailability of P can be regulated by both direct and indirect factors. When TiO_2_ and Fe_3_O_4_ ENPs were added to a sandy-loam soil, the phytoavailability of the P bound to the rhizosphere increased. The results were due to the acidification produced by the exudation of organic acids of *Lactuca sativa* roots Zahra et al. [108]. A similar mechanism was proposed for an increase in the concentration of available phosphate in the presence of Fe ENPs [99] and CeO_2_ ENPs [47]. According to the report by Feng et al. [109], composites of CeO_2_ ENPs-functionalized maize straw biochar (CeO_2_-MSB) decreased the total phosphorus (TP) concentration of surface water by 27.33% and increased the TP content of the upper soil layer by 7.22%. Although this indeed caused an increase in P adsorption, it could be interesting to establish that the interaction between P and soil caused an increase in the height of the rice plant and the foliar area. Therefore, CeO_2_-MSB could be used to reduce the risk of P loss from the surface of rice fields.

## 4. Conclusions and Perspectives

Metal and metallic oxide ENPs deposition/accumulation in soils will increase over time. In general, we have determined that ENPs can compact the particles, helping to improve their rigidity, as well as causing changes in pH, EC, Eh, and SOM. These results depended on the soil properties and the doses, concentrations, and types of ENPs. Furthermore, the presence of ENPs or the cations released from them in soils can interact with nutrients, forming complexes or precipitates and modifying their availability in the soil solution. Research into the impacts of ENPs on physical and chemical soil properties is still in its initial stage. For this reason, future studies should investigate not only the advantages of applications of metal and metallic oxide ENPs in agricultural systems but also their risks and disadvantages, like their impact on soil health and quality, considering abiotic properties as well as microorganisms and plants in the short and long term.

## Figures and Tables

**Table 1:** Effect of metal and metallic oxide engineered nanoparticles (ENPs) on some soil physical and chemical properties.

ENPs Type	Concentration	Type or Place of Soil	Duration	Remarks	Reference
	**Physical properties**				
Pt	0.1–1000 μg g^−1^	Clay-free organic sapric histosol	5 weeks	Increase structural rigidity of SOM and aliphatic crystallites content; decrease in the enthalpy of evaporation of water in the SOM	[24]
γ-Al_2_O_3_ and CuO	0.05–0.3% γ-Al_2_O_3_, 0.15–0.7% CuO	Selangor, Malaysia	10 days	Reduction of the swelling stress and the shrinkage stress of the soil; decrease in hydraulic conductivity and density	[32]
Fe_3_O_4_ and MgO	1, 3, 5% (*w/w*)	Agricultural land in Hamedan, Iran	100 days	The bulk density of the soil increases with the dose of Fe_3_O_4_ ENPs but decreases with MgO ENPs	[33]
SiO_2_ and Zn	50 mg L^−1^ of Zn or 2.5 mg L^−1^ of SiO_2_	El-Serw Agricultural Research Station	7 weeks	Increase in the hydraulic conductivity of the soil; decrease in bulk density	[34]
Fe	1, 4, 7, 10 g L^−1^	Oxisol	-	Concentrations <4 g L^−1^ do not affect the natural hydraulic conductivity of the soil. However, higher concentrations reduced the hydraulic conductivity value	[35]
Ag coated with polyvinylpyrrolidone (PVP) and citrate	2.5, 5.0, 10 mg L^−1^	Red Soil	-	The surface coatings of Ag ENPs block the solid phase sites promoting the transport of the ENPs	[36]
	**Chemical Properties**				
Ag	20, 25, 50 and 100 mg kg^−1^	Alluvial soil of Tezpur, India	60 days	Increase in the CEC, pH soil, and N and P bioavailability	[10]
CuO and Fe_3_O_4_	1 or 5% (*w/w*)	Red Sandy clay loam Mediterranean soil and Rendzina soil	24 h	Fe_3_O_4_ ENPs catalyze the oxidation of organic pollutants in aqueous suspensions, inducing changes in SOM	[23]
TiO_2_, ZnO and CuO	50, 100 and 500 mg kg^−1^	Paddy soils	90 days	Increase of soil pH, Eh, and EC in flooding-drying process	[28]
Fe	0.1, 1, 10 mg g^−1^	Hangzhou, Taizhou, Haikou, Kunming, Honghe, Chifeng, Puer, and Yingtan	90 days	ENPs promote aromatic carbon sequestration and decrease the Eh of the soil. The impact of ENPs on soil pH, EC, ζ potential, dissolved organic carbon (DOC), and enzyme activity is dependent on the soil type and soil moisture content	[37]
Fe	10 mg g^−1^	Silt loam soil	14 days	Decrease in Eh and increase in soil pH	[38]
Fe	28–36 mg g^−1^	Acidic and calcareous	30 days	Modification of pH values depending on the buffering capacity of the soil; increased EC and water retention capacity of soils	[39]
Fe	1, 5, and 10% (*w/w*)	From El Terronal and Asturias.	72 h	No effect on soil pH and EC	[40]
TiO_2_	1 and 500 mg kg^−1^	Sandy-loam, loam and silty-clay	90 days	Low doses of ENPs decrease the mineralization of C in a clay-silty soil	[41]
Fe_3_O_4_	1000 and 2000 mg L^−1^	Loamy	30 days	Increase the CEC and total P content and P extractable	[42]
Fe	0.10–2.0 g L^−1^	-	48 h	The residual DOM has a higher reduction capacity, % mineralization and photodegradation after the adsorption of ENPs	[43]
CuO	50,100, 500 and 1000 mg kg^−1^	Hangzhou	88 days	High concentrations of ENPs decrease the Eh but improve EC; increased soil pH; increased phyto-availability of Cu in the soil	[44]
TiO_2_	50 and 100 mg kg^−1^	Seoul	40 days	Increase in EC and decrease in pH of the rhizosphere; improves P dissolution	[45]
CuO	10, 100, and 1000 mg kg^−1^	Paddy soils	90 days	Increased degradation and mineralization of OM; increased in soil pH	[46]
CeO_2_ and TiO_2_	500 mg kg^−1^	Southern Australian soils	260 days	Both ENPs alter the mineralization of organic N and/or the nitrification rates of the soil due to the catalytic and/or antimicrobial properties of the ENPs; increase in the phyto-availability of P and Zn in soils	[47]
ZnO	2.5 mg kg^−1^	Inceptisol	60 days	Decrease in soil pH and SOC; increased EC and P available	[48]
SiO_2_	4.5 mg L^−1^	Wuhan, Chongqing, and Qianjiang	24 h	Decreased mobility of pesticides in soils, although this effect varies with the composition of the soil	[49]
ZnO	100 and 1000 mg kg^−1^	Agronomy farm of Faisalabad	64 days	Increase in soil pH and C mineralization	[50]
Fe, Fe_3_O_4_, and Fe_2_O_3_	2 to 6 g kg^−1^	Udic Ferrosols and Anthrosol	60 days	Fe ENPs increase in DOC and available NH_4_^+^-N but decrease available phosphorus (AP), while Fe_3_O_4_ and Fe_2_O_3_ ENPs slightly reduce soil pH and decrease available NH_4_^+^-N and AP	[51]
CuO	10 and 100 mg kg^−1^	Sandy soil	31 days	Increase soil pH	[52]
ZnO	1.0 and 20.0 mg	Agricultural-clay soil and peaty soil	4 weeks	Decrease in the content of Al, Ca, Cu and Mg in the soil	[53]

## Data Availability

Not applicable.
